# Extensively Drug-Resistant *Streptococcus pneumoniae*, South Korea, 2011–2012

**DOI:** 10.3201/eid2005.131371

**Published:** 2014-05

**Authors:** Sun Young Cho, Jin Yang Baek, Cheol-In Kang, So Hyun Kim, Young Eun Ha, Doo Ryeon Chung, Nam Yong Lee, Kyong Ran Peck, Jae-Hoon Song

**Affiliations:** Samsung Medical Center, Sungkyunkwan University School of Medicine, Seoul, South Korea (S.Y. Cho, C.-I. Kang, Y.E. Ha, D.R. Chung, N.Y. Lee, K.R. Peck, J.-H. Song);; Asia Pacific Foundation for Infectious Diseases, Seoul (J.Y. Baek, S.H. Kim, J.-H. Song)

**Keywords:** Streptococcus pneumoniae, extensively drug-resistant, bacteria, South Korea, antimicrobial drug resistance

## Abstract

To better understand extensively drug resistant *Streptococcus pneumoniae*, we assessed clinical and microbiological characteristics of 5 extensively drug-resistant pneumococcal isolates. We concluded that long-term care facility residents who had undergone tracheostomy might be reservoirs of these pneumococci; 13- and 23-valent pneumococcal vaccines should be considered for high-risk persons; and antimicrobial drugs should be used judiciously.

During the past 2 decades, multidrug-resistant *Streptococcus pneumoniae* has spread worldwide (*1*,*2*). Recently we reported a case of bacteremic pneumonia caused by extensively drug-resistant (XDR) *S. pneumoniae* (*3*). Five additional XDR pneumococcal isolates subsequently were identified in our hospital in South Korea. In an attempt to better understand the epidemiologic and clinical aspects of XDR *S. pneumoniae*, we investigated clinical and microbiological characteristics of these cases of XDR *S. pneumoniae*.

## The Study

We reviewed the database of the clinical microbiology laboratory at Samsung Medical Center (SMC, Seoul, South Korea) for XDR *S. pneumoniae* isolates obtained during 2011–2012. XDR *S. pneumoniae* was defined as nonsusceptibility to at least 1 agent in all antibacterial drug categories except vancomycin and linezolid.

Among the 510 *S. pneumoniae* isolates (319 in 2011 and 191 in 2012), we identified 5 XDR pneumococcal isolates from 5 (1.2%) patients. The following data were obtained for the patients: age, sex, date of isolation, prior hospitalization, residence in long-term care facilities (LTCFs), underlying diseases, site of bacterial isolation, status of bacterial isolation (infection or colonization), prior prophylaxis and therapy with antibacterial drugs, and outcome.

In vitro antimicrobial susceptibility tests of pneumococcal isolates were retested by the broth microdilution method according to Clinical and Laboratory Standards Institute guidelines (*4*). Antimicrobial classes tested included penicillins (penicillin), cephalosporins (ceftriaxone), macrolides (erythromycin, clarithromycin), quinolones (levofloxacin), clindamycin, trimethoprim/sulfamethoxazole, carbapenems (imipenem), tetracyclines (tetracycline, tigecycline), glycopeptides (vancomycin), and linezolid. Interpretive criteria for susceptibility were those indicated in a Clinical and Laboratory Standards Institute document (*5*). Serotypes of *S. pneumoniae* were determined by the capsular puellung method with commercial antiserum (Statens Serum Institut, Copenhagen, Denmark) as recommended by the manufacturer. To investigate the molecular characteristics of XDR *S. pneumoniae*, we performed multilocus sequence typing (MLST) and pulsed-field gel electrophoresis (PFGE) as described (*6*,*7*). Also, all isolates were subjected to PCR to detect quinolone resistance–determining regions and macrolide resistance genes as described (*8*,*9*).

Four of 5 XDR *S. pneumoniae* isolates had been isolated from respiratory tract specimens (e.g., sputum), and 1 isolate had been recovered from blood (Table 1). Mean age (± SD) of patients was 71.8 (± 16.9) years. Three patients were admitted from 3 different LTCFs, and 2 patients were referred to our hospital from other acute care hospitals. The most common underlying diseases were neurologic disorders, such as cerebrovascular disease and motor neuron disease (3 patients), followed by diabetes mellitus (2 patients) and solid tumor (1 patient) (1 patient had both motor neuron disease and diabetes mellitus). Four patients underwent tracheostomy because of respiratory problems, such as neurologic disease and progression of underlying diseases, and had multiple episodes of aspiration pneumonia. All patients had received antibacterial drug therapy within the past 3 months. The most frequently used antibacterial drugs were fluoroquinolones (4 patients) and piperacillin–tazobactam (3 patients). Four of the 5 patients did not have clinical evidence of infection with XDR *S. pneumoniae*; bacteremia from this pathogen developed in the remaining patient. The patient with bacteremia was admitted to the emergency department of SMC with fever. Three weeks before admission, nephrotic syndrome had been diagnosed, and the patient had received immunosuppressant therapy with methylprednisolone and cyclophosphamide. Soon after admission, septic shock developed, and ciprofloxacin was administered because urinary tract infection was suspected. On hospital day 4, after the blood culture results showed XDR *S. pneumoniae*, vancomycin was started. Despite the administration of vancomycin for 7 days, the patient died from progression of septic shock. Because she had difficulty in expectorating sputum, we could not obtain adequate sputum samples despite sputum induction. However, given the clinical symptoms consistent with pneumonia and a chest radiograph that demonstrated pulmonary infiltrate, the most likely source of bacteremia appears to be pneumonia caused by *S. pneumoniae*.

**Table 1:** Clinical features and outcomes of 5 patients with extensively drug-resistant *Streptococcus pneumoniae* from Samsung Medical Center, Seoul, South Korea, 2011–2012*

Strain no.	Date of isolation	Sex/age, y	Prior hospitalization	Underlying disease	Comorbid condition	Specimensource	Infection	Empirical use of antimicrobial drugs	Definite antimicrobial drugs	Outcome
SMC1101–114	2011/01/02	F/64	Hospital	CV disease	Tracheostomy	Sputum	Colonization	NA	NA	NA
SMC1101–127	2011/01/10	M/46	LTCF	Solid tumor	Tracheostomy,steroid	Sputum	Colonization	NA	NA	NA
SMC1103–146	2011/03/07	F/86	Hospital	CV disease, renal disease	Immunosuppressant, steroid	Blood	Pneumonia,bacteremia	Ciprofloxacin	Vancomycin	Death
SMC1105–235	2011/05/14	F/85	LTCF	DM	Tracheostomy	Sputum	Colonization	NA	NA	NA
SMC1108–054	2011/07/29	M/78	LTCF	Motor neuron disease, DM	Tracheostomy	Sputum	Colonization	NA	NA	NA

*LTCF, long-term care facility; NA, not applicable; CV, cerebrovascular; DM, diabetes mellitus; SMC, Samsung Medical Center.

The 5 pneumococcal isolates reported in this study were nonsusceptible to all tested antimicrobial agents except tigecycline, vancomycin, and linezolid (Table 2). The serotypes of isolates were 11A (3 isolates) and 13/28 (1 isolates); 1 isolate was not typeable. MLST demonstrated that 4 isolates were sequence type (ST) 8279, a double-locus variant of ST156 closely related to the pneumococcal Spain9V-3 international clone, and 1 isolate was ST3598. PFGE patterns showed close genetic relatedness among 3 isolates and 2 isolates (94.7% and 84.2% genetic relatedness, respectively) (Figure). However, SMC 1205–093, reported in 2012, had a different PFGE pattern from the other 5 isolates. Sequence analysis of quinolone resistance–determining regions revealed the same mutation pattern in these 5 isolates: Ser81-Phe in *gyrA*, Lys137-Asn in *parC*, and Ile460-Val in *parE*. In addition, as a macrolide resistance determinant, only *erm*(B) gene was detected by PCR in all isolates.

**Table 2:** Antimicrobial susceptibilities of 5 extensively drug-resistant *Streptococcus pneumoniae* isolates, Seoul, South Korea, 2011–2012*

Strain no.	MIC, μg/mL											
	LZD	TGC	VAN	PEN	TMP/SXT	CLI	CRO	LVX	ERY	CLR	TET	IPM
SMC1101–114	0.5	<0.03	0.5	8	32/608	>32	16	16	64	16	32	2
SMC1101–127	0.5	<0.03	0.25	8	32/608	>32	64	16	128	>32	32	2
SMC1103–146	0.5	<0.03	0.25	8	32/608	>32	64	16	128	>32	32	2
SMC1105–235	0.5	<0.03	0.25	8	32/608	>32	32	16	128	>32	32	2
SMC1108–054	0.5	<0.03	0.5	8	32/608	>32	64	16	128	>32	16	2

*LZD, linezolid; TGC, tigecycline; VAN, vancomycin; PEN, penicillin; TMP/SXT, trimethoprim/sulfamethoxazole; CLI, clindamycin; CRO, ceftriaxone; LVX, levofloxacin; ERY, erythromycin; CLR, clarithromycin; TET, tetracycline; IPM, imipenem; SMC, Samsung Medical Center.

**Figure:**
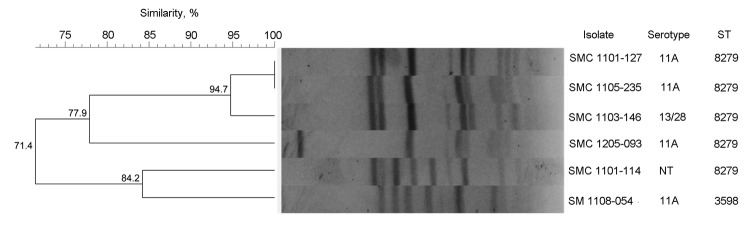
Dendrogram of pulsed-field gel electrophoresis patterns showing the genetic relatedness of extensively drug-resistant pneumococcal isolates from patients in South Korea, 2011–2012 (including SMC 1205–093, previously reported in 2012). The corresponding serotype and sequence type (ST) for each isolate are listed on the right side of the dendrogram. SMC, Samsung Medical Center.

## Conclusions

We reassessed isolates obtained during 2011–2012 and identified 5 pneumococcal isolates that were not susceptible to at least 1 agent in all antimicrobial classes tested (penicillins, cephalosporins, macrolides, quinolones, clindamycin, tetracyclines, trimethoprim/sulfamethoxazole and carbapenems) except vancomycin and linezolid. Of the 5 patients, 1 with bacteremia died despite treatment.

Our findings have several clinical implications. First, the cases reported here showed that LTCF residents who had undergone tracheostomy might be a reservoir of XDR pneumococci. Also, our data documented a genetic relationship between XDR pneumococcal isolates shown by MLST and PFGE, which suggests that specific serotypes and resistant clones are spreading within certain LTCFs. Considering the characteristics of LTCF residents, the spread of XDR *S. pneumoniae* among these patients can lead to considerable illness and to death.

Second, this study indicated that 3 of the 5 XDR isolates were serotype 11A, which were included in 23-valent pneumococcal polysaccharide vaccine (PPV23) but not in the 7-valent pneumococcal conjugate vaccine (PCV7) and the 13-valent pneumococcal conjugate vaccine (PCV13). Although we were unable to determine the status of pneumococcal vaccination of the patients reported here, given the very low rates of PPV23 vaccination in Korea (<5%), these patients were likely to be unvaccinated (*10*). Among pneumococcal isolates collected from patients with respiratory tract infections in South Korea, the prevalence rate of serotype 11A was 7.6%, and these serotype 11A isolates showed a high prevalence of multidrug-resistance (65.2%) (*11*). In addition, after the introduction of PCV7 in Korea, nonvaccine serotypes (7C, 11A, 15A, 16F, and 23A) have increased in levofloxacin-nonsusceptible pneumococcal isolates (*12*). PCV13 was approved for all adults >50 years of age in 2012 and is now widely used in South Korea. If PCV13 is routinely used instead of PPV23 for adults, non-PCV13 serotypes with multidrug resistance potentially could emerge. Therefore, in adults at risk for MDR pneumococcal infection, including previous use of antibacterial drugs, LTCF residence, and multiple comorbidities, administration of 2 pneumococcal vaccines (PCV13 and PPV23) should be considered.

Third, antibacterial drugs should be used judiciously. In particular, given the increasing evidence that prior use of fluoroquinolones may be a major risk factor for fluoroquinolone resistance among pneumococci, the use of fluoroquinolones should be restricted to patients at increased risk for MDR pneumococcal infection (*13*,*14*).

Although still rare, the emergence of XDR pneumococci has become challenging for clinicians and a real threat to public health. More information about the emergence and spread of this XDR strain is necessary to prevent its spread, and continuous surveillance of XDR *S. pneumoniae* is strongly warranted.
